# Second cancer incidence and cancer mortality among chronic lymphocytic leukaemia patients: a population-based study

**DOI:** 10.1038/bjc.2011.313

**Published:** 2011-08-16

**Authors:** J A Royle, P D Baade, D Joske, J Girschik, L Fritschi

**Affiliations:** 1 Western Australian Institute for Medical Research, University of Western Australia, Hospital Avenue, Nedlands WA 6009, Perth, Western Australia, Australia; 2 Viertel Centre for Research in Cancer Control, Cancer Council of Queensland, PO Box 201, Spring Hill, Brisbane QLD 4001, Queensland, Australia; 3 School of Public Health, Queensland University of Technology, Kelvin Grove, Brisbane QLD 4059, Queensland, Australia; 4 Sir Charles Gairdner Hospital, Hospital Avenue, Nedlands WA 6009, Perth, Western Australia, Australia

**Keywords:** chronic lymphocytic leukaemia, second cancer, melanoma, skin cancer, mortality

## Abstract

**Background:**

Patients with chronic lymphocytic leukaemia (CLL) are known to have increased risks of second cancer. The incidence of second cancers after CLL has not been reported in detail for Australia, a country with particularly high levels of ultraviolet radiation (UVR).

**Methods:**

The study cohort comprised of all people diagnosed with a primary CLL between 1983 and 2005 in Australia. Standardised incidence ratios (SIRs) and standardised mortality ratios (SMRs) were calculated using Australian population rates.

**Results:**

Overall, the risk of any second incident cancer was more than double that of the general population (SIR=2.17, 95% confidence interval (CI)=2.07, 2.27) and remained elevated for at least 9 years after CLL. Risks were increased for many cancers, particularly melanoma (SIR=7.74, 95% CI=6.85, 8.72). The risk of melanoma increased at younger ages, but was constant across >9 years of follow-up. Chronic lymphocytic leukaemia patients also had an increased risk of death because of melanoma (SMR=4.79, 95% CI=3.83, 5.90) and non-melanoma skin cancer (NMSC; SMR=17.0, 95% CI=14.4, 19.8), suggesting that these skin cancers may be more aggressive in CLL patients.

**Conclusion:**

We speculate that a shared risk factor, such as general immune suppression, modulated by UVR exposure may explain the increased risk of melanoma and NMSC in CLL patients.

Chronic lymphocytic leukaemia (CLL) is a malignancy of unknown aetiology, and is characterised by the accumulation of B lymphocytes in the peripheral blood, bone marrow and secondary lymphoid organs (Ghia *et al*, 2007). The condition develops slowly and the treatment may not be required for months or years. Although there is no ‘cure’ as such, it is not rapidly fatal thus there are many people living with this disease. Approximately, 1000 new cases are diagnosed in adults in Australia each year. Chronic lymphocytic leukaemia is more common in men than in women, with a sex ratio of about 1.5. (AIHW and AACR, 2008).

There is a general consensus in the literature that the risk of developing a second cancer is increased in those living with CLL (Wiernik, 2004). Two recent reviews found that the second cancers with the highest increased rate included melanoma, non-melanoma skin cancer (NMSC), lung, other respiratory tract, oral cavity and pharynx, prostate, kidney and lymphoma (Wiernik, 2004; Molica, 2005). Several mechanisms have been discussed for the association between CLL and second cancers, including disease- and treatment-related immunosuppression and shared risk factors. Furthermore, there are reports that squamous cell carcinoma (SCC) of the skin is particularly aggressive in patients with CLL (Hartley *et al*, 1996; Wiernik, 2004). A study we recently published showed about 10% of patients who died from SCC of the skin had a concurrent diagnosis of CLL, and this proportion is much higher than would be expected in the usual population (Girschik *et al*, 2008).

We recently published a study examining the risk of second cancers after all lymphohematopoietic neoplasms (LHNs) in Australia (Royle *et al*, 2010). Overall, patients with an LHN had nearly twice the risk of developing a second cancer, particularly melanoma, compared with the general Australian population. Australia has different patterns of cancers compared with other countries for which second cancer risks have been published, including the highest incidence of skin cancers in the world (Parkin *et al*, 2003). This study examines the risk of second cancers after CLL, as the most common subgroup of LHNs and a chronic disease. We used population-based data to characterise the site-specific risks of second cancers after CLL and examine cancer mortality among CLL patients. NMSC was a focus of this study, and it is not reportable in Australia, therefore we also examined mortality from cancer (including NMSC) in patients with CLL.

## Methods

The study cohort comprised of all people diagnosed with a primary CLL (ICD-O-3 code M9823/3) between 1 January 1983 and 31 December 2005 in Australia. Data on cases of CLL were obtained from the National Cancer Statistics Clearing House (NCSCH), which is maintained by the Australian Institute of Health and Welfare (AIHW). The NCSCH receives data from individual state and territory (population based) cancer registries on all cancers diagnosed in residents of Australia (except NMSC). The notifications to these registries are required by the law, and the registration is thought to be virtually complete.

Incidence data for the second cancers were also sourced from the NCSCH. We examined the risk from all invasive cancers (ICD code C0–C96) as well as major subgroups providing there were adequate numbers of cases in each group (total *n*⩾5, incident second cancers or ⩾20 deaths in each cancer group).

Cancer mortality data was sourced from the National Death Index. Codes for mortality data up to and including 1996 used the ICD-9 coding classification, whereas those from 1997 and beyond used the ICD-10 classification. The cohort of CLL survivors was linked to the mortality records by the AIHW. For the purpose of this study, people who were not known to have died by 31 December 2005 were assumed to be alive at that date.

The comparison group was the population of Australia. Estimated resident population data, and the Australian incidence data on second cancers were sourced from the NCSCH.

### Statistical analysis

Data extractions and analysis were conducted using SAS (SAS Institute Inc., Cary, NC, USA) and Stata (StatCorp, College Station, TX, USA). Initial data extraction was conducted by AIHW. The relevant programming codes for the analyses were sent electronically to AIHW staff who submitted the programmes and then returned the aggregated results to the investigators. This ensured that no potentially identifying information was released outside the AIHW. The study was approved by the Human Research Ethics Committee of The University of Western Australia.

For the incident cancer study, person-years at risk were accumulated for each person in the CLL cohort from the date of diagnosis of CLL up to the date of diagnosis of second cancer, date of death or 31 December 2005, whichever came first. To reduce the effect of detection bias, cancers present concurrently at the time of CLL diagnosis (rather than subsequent to CLL diagnosis), and secondary cancers diagnosed within 3 months of the first cancer were excluded (Schollkopf *et al*, 2007). The expected number of second cancers was calculated by applying the 5-year age, decade of diagnosis and sex-specific incidence rate for each condition in Australia to the person-years at risk among the CLL cohort. Indirectly, age-standardised incidence ratios (SIRs) were calculated for each site of cancer as the ratio of the observed number of cases to the expected number of cases. In all, 95% confidence intervals (95% CIs) for these SIRs were calculated assuming a Poisson distribution for the observed cases. Cancer types with less than five observed cases were excluded from these results.

For the mortality study, similar processes were followed. Person-years at risk were accumulated for each person in the CLL cohort from the date of diagnosis up to the date of death or 31 December 2006, whichever came first. As for incidence, the expected number of cause-specific deaths was calculated by applying the 5-year age, decade of death and sex-specific mortality rate for each cause in Australia to the person-years at risk among the CLL cohort. Indirectly, age-standardised mortality ratios (SMRs) were calculated by dividing the observed number of deaths by the expected number of deaths. Those who died within 3 months of CLL diagnosis were excluded from the cohort.

## Results

In the incidence cohort (followed to the end of 2005), 13 580 patients were diagnosed with CLL between 1983 and 2005 in Australia (Table 1). The CLL patients were followed for a total of 75 878 person-years at risk (median length of follow-up 4.3 years) during which 1793 second cancers occurred (13.1%). In the mortality cohort (followed to the end of 2006), 13 734 of the CLL patients were included and were followed for 84 482 person-years at risk (median length of follow-up 4.9 years). There were 8246 deaths and 64.8% of those deaths were due to cancer. For both the cohorts, the male-to-female ratio was 1.5:1 and the median age at CLL onset was 69.

The overall risk of second primary cancer at specific sites after CLL diagnosis is presented in Table 2. The risk of second malignancy overall for CLL patients was increased (SIR=2.17, 95% CI=2.07, 2.27) and remained elevated for at least 9 years after CLL. Significant excesses were seen for cancers of the lip, oral cavity and pharynx, larynx, colorectum, lung, melanoma, connective tissue and peripheral nerves, female breast, uterine corpus, prostate, kidney, bladder, eye, thyroid and Hodgkin's disease (HD), whereas there was a significant decreased risk of being diagnosed with non-Hodgkin's lymphoma (NHL) or myeloid leukaemia. Both males and females had a similar risk pattern at all sites when compared with the sex-specific population.

Following CLL, significant increased risks of second cancer were observed over all age groups and follow-up times for cancers of the colorectum, lung, melanoma, prostate and kidney (Table 3). Elevated risks at younger ages were generally observed for most of these cancers.

Death from cancer represented 64.8% of the mortality among the CLL cohort; lung cancer and skin cancer accounting for the greatest cancer mortality not including lymphoproliferative neoplasms (Table 4). The age SMRs for all causes of death are reported in Table 5. The overall mortality among CLL patients was 2.5 times higher than the general population (SMR=2.55, 95% CI=2.49, 2.60). As expected, the risk of dying from a lymphoproliferative neoplasm, including CLL, was ∼50 times higher for CLL patients compared with the general population. After excluding deaths from lymphoproliferative neoplasms, CLL patients still had a 72% increased risk from death due to cancer. In particular, the CLL cohort had significantly increased mortality from cancers of the colorectum, lung and skin (both NMSC and melanoma). Death due to of NMSC and melanoma had the highest SMR of all causes. Males and females had a similar mortality pattern for all causes of death.

## Discussion

We observed a doubling in risk of second cancer in patients with CLL, consistent with previous observations (reviewed in Wiernik, 2004 and Molica, 2005). The increased cancer risk was observed in both men and women and across all age groups, and remained relatively constant over time since diagnosis of CLL. Similar findings were reported in a recent Danish study (Schollkopf *et al*, 2007), which assessed the risk of second cancers among 12 373 patients diagnosed with CLL in Denmark between 1943 and 2003. Compared with the current study, they observed a slightly lower increased risk of second cancers overall (SIR=1.59, 95% CI=1.50–1.69); however, consistent with our findings, the increased risk persisted >9 years after CLL diagnosis.

Detection bias may be an explanation for these results. To address this, we excluded any second cancers diagnosed within three months of the first cancer (Schollkopf *et al*, 2007). Although CLL is often managed as a chronic disease, with long term follow-up, it would be less likely that a physician would do a comprehensive examination at follow-up visits than in the early stages of disease. However, patients with CLL will have contact with the health system for many years after diagnosis and may bring symptoms of a second cancer to their physician earlier than usual. If detection bias is present, it may increase the risk of incident cancer, especially those for which early detection is possible (such as prostate cancer), but perhaps decrease the risk of death from cancer (especially cancers with treatment available such as NMSC and breast cancer).

A second possible cause of the observed increased risk of developing a second cancer is that the second cancer is an effect of treatment of the CLL. However, two recent studies found little evidence to suggest an association between treatment for CLL/small lymphocytic leukaemia and subsequent development of second cancers (Callea *et al*, 2006; Tsimberidou *et al*, 2009). The treatment for CLL for many years and during a large portion of the time of this study was simple alkylator-based therapy with oral chlorambucil, which is not in itself very immunosuppressive. Chlorambucil is considered leukemogenic, but this applies particularly to the treatment of myeloproliferative disorders, such as polycythaemia rubra vera, and essential thrombocythemia where the marrow already has acquired a hyperplastic state. The first-line treatment of CLL is tending to change (Carney and Mulligan, 2009) towards more aggressive first-line therapy with a purine analogue/alkylator combination, flubarabine, cyclophosphamide and sometimes rituximab. This combination is very immunosuppressive with high rates of infection and cytopenias. It may be that we will see secondary marrow damage with this combination in future years.

The third possible cause of an increased risk of second cancer in CLL patients is that there are common risk factors between the cancers (including environmental exposures or heritable factors). Cancers that share a common risk factor are likely to be consistent across time since first diagnosis. In our study, excess risk for second cancers occurred in all time periods since diagnosis, suggesting a shared risk factor (Morton *et al*, 2010).

For >20 years, it has been suggested that the chronic immunosuppression in CLL patients may have a role in their subsequent risk for skin cancer (Greene and Wilson, 1985). Chronic immunosuppression has been linked to both skin cancer and CLL. Increased risk of melanoma has been reported following HD, NHL, CLL, renal transplantation, immunosuppressive therapy and among those with genetically determined immunodeficiency (McKenna *et al*, 2003). In addition, small increased risks of CLL have also been linked with chronic infectious disorders, autoimmune diseases, allergic disorders and appendectomy in some studies, although results have been inconsistent (Linet *et al*, 2006). Exposure to ultraviolet radiation (UVR) may also have a role in the association between CLL and cancer of the skin and lip (Adami *et al*, 1995; Dong and Hemminki, 2001; Schollkopf *et al*, 2007). Information on UVR exposure was not available for our analysis; however, it is now commonly accepted that lifetime exposure to UVR through chronic sun exposure is causal in the development of SCCs, and, for melanoma, high intermittent exposures during childhood and adolescence are considered important (Adami *et al*, 1995; English *et al*, 1997; Mayne *et al*, 2006). Furthermore, skin cancer prevention measures for transplant recipients includes protection against solar and artificial UVR suggesting that UVR exposure may modulate the risk of skin cancer already increased by a common mechanism such as general immune suppression (Reichrath and Nurnberg, 2008).

Melanoma was one of the most frequent cancer sites in our study, with 272 observed cases corresponding to more than seven-fold increased risk. The risk was increased at younger ages, but was constant across >9 years of follow-up. Chronic lymphocytic leukaemia patients also had a nearly five-fold increased risk of death because of melanoma. In addition, there was a considerable increase in the risk of death from NMSC, suggesting these skin cancers may be more aggressive in CLL patients given the typically benign course of these malignancies. As both NMSC and melanoma are rarely fatal if detected early, the high mortality from skin cancer provides evidence consistent with the hypothesis that detection bias is not the cause of the higher incidence rates of melanoma. Increased risks of melanoma and skin cancer in CLL patients have been described in other population-based studies (Dong and Hemminki, 2001; Hisada *et al*, 2001; McKenna *et al*, 2003; Schollkopf *et al*, 2007). Hisada *et al.* (2001) found a statistically significant increased risk of malignant melanoma (SIR=3.18) after CLL. Squamous cell carcinoma of the skin was also found to be increased in patients with a lymphoid leukaemia (70% CLL) in Sweden (SIR=5.3, 95% CI=4.4–6.3; Dong and Hemminki, 2001). Similarly, McKenna *et al.* (2003) reported an increased incidence of malignant melanoma after a diagnosis of CLL (SIR=2.3) and vice versa (SIR=2.3), suggesting a common risk factor. We also found a seven-fold increased risk of lip cancer after CLL (SIR=6.82, 95% CI=4.97, 9.12). Australia has one of the highest rates of lip cancer, and SCC, generally, accounts for 95% of those cancers (Parkin *et al*, 2003; Mayne *et al*, 2006). The risk of lip cancer was more apparent among CLL cases with a younger age at onset and increased with time. Details of lip cancer have been reported in only one study of CLL patients and no increase was noted (Schollkopf *et al*, 2007); however, lip cancer has been linked to NHL and HD patients (Swerdlow *et al*, 2000; Brennan *et al*, 2005).

A large excess of cancer of the eye was observed in our study (SIR=5.98, 95% CI=2.73, 11.4). Although we were unable to confirm the tumour type, ocular melanoma accounts for ∼80% of malignancies of the eye (Gruber and Armstrong, 2006). A moderate increased risk of cancer of the eye (all ocular melanoma) was also observed in the SEER database (Hisada *et al*, 2001); however, the estimate was not statistically significant.

Our study found that CLL patients had a higher risk of smoking-related cancers including the oral cavity and pharynx, larynx, lung, kidney and bladder. Several previous studies have observed excess risks of lung cancer following CLL diagnosis (Greene and Wilson, 1985; Travis *et al*, 1992; Schollkopf *et al*, 2007). Smoking is the cause of 85–90% of lung cancers (Spitz *et al*, 2006). A review in 2002 found little evidence for an association between smoking and CLL (Kuper *et al*, 2002), although one study since then suggested a small statistically nonsignificant increase in risk for smoking >2 years before CLL diagnosis (Richardson *et al*, 2008). Smoking information was not available in our register-based study; however, it seems plausible that the carcinogenic effects of smoking may be amplified by treatment and/or immune deficiency (van Leeuwen *et al*, 1995; Swerdlow *et al*, 2001).

The risk of thyroid cancer, colorectal cancer, uterine cancer and cancer of the connective tissue and peripheral nerves were also increased in our study. Schollkopf *et al.* (2007) reported similar results for thyroid cancer and cancer of the connective tissue and peripheral nerves, but there is little other evidence regarding these cancers.

A slight increase in the risk of female breast cancer was observed in our study after CLL (SIR=1.93, 95% CI=1.55, 2.39). Although a recent population-based study (Schollkopf *et al*, 2007) reported a decreased risk of breast cancer after CLL, other evidence supports our finding that CLL patients may have increased risk of breast cancer. A previous review (Molica, 2005) on second neoplasms in CLL described that breast cancer can be diagnosed with an increased frequency among first-degree relatives of patients with CLL, and that CLL and breast cancer share common cytogenetic aberrations. Furthermore, oestrogen receptors have been described in some CLL cases who achieved a reduction of leukaemic disease with tamoxifen therapy (Melo *et al*, 1990). Alternatively, surveillance bias may explain our finding, as mortality from breast cancer was not increased in CLL patients.

Our study found a small increased risk for prostate cancer after CLL (SIR=1.94, 95% CI=1.71, 2.18). There is evidence to suggest an association between CLL and prostate cancer (Molica, 2005). Deletion of chromosome 13q14, a common finding in B-cell CLL, is also found in some patients with prostate cancer (Sossey-Alaoui *et al*, 2002; Hammarsund *et al*, 2004). Furthermore, it is important to note that 1–2% of patients who undergo radical prostatectomy for prostate cancer have indolent CLL lymph node involvement (Terris *et al*, 1997; Weir and Epstein, 2003). However, CLL patients were at a decreased risk of prostate cancer death (SMR=0.64, 95% CI=0.41, 0.95). This could be because of the fact that these cancers are being diagnosed at an earlier stage due to CLL patients having more frequent contacts with the health system. The lower mortality from prostate cancer in the CLL patients supports this explanation.

We observed an increased risk of HD (SIR=7.98, 95% CI=3.98, 14.3) in the CLL cohort, whereas risk of NHL and myeloid leukaemia were decreased (SIR=0.28, 95% CI=0.10, 0.62 and SIR=0.40, 95% CI=0.13, 0.92, respectively). The interpretation of these results should be carried out with caution given that the diagnosis and notification of these disorders secondary to CLL may be biased. However, the excess of HD could be because of the evolvement of CLL into aggressive large-cell lymphoma (Richter's transformation/syndrome), often resembling HD (Robak 2004; Schollkopf *et al*, 2007).

Overall, CLL patients were at about 2.5-fold mortality compared with the general population. After excluding the 50% of the deaths that were attributed to a LHN, deaths due to lung cancer and skin cancer were the largest contributors to the cancer deaths.

Our study has both strengths and weaknesses. To our knowledge, ours is the first study to investigate the association of CLL and second cancers in the Australian population. This study was based on the Australian Cancer Database records of all cases of cancer in Australia since 1982 and information available on vital status of these patients by linkage with the National Death Index. The large dataset enabled us to analyse site-specific risk of second cancers following CLL, as well as to describe age and latency patterns for second cancer risk. We were able to adjust the SIRs for decade of diagnosis, which may be a potential confounding factor.

Limitations of our study include the lack of information on NMSC incidence (it is not a reportable cancer in Australia), as well as the missing data on treatment and smoking habits. Furthermore, cause of death was based on death certificate data; and, therefore, misclassification of cause of death is a possibility. A previous Australian study reported that 23% of NMSC deaths were misclassified (Rosenblatt and Marks, 1996), which may account for the extreme NMSC SMR of 17.0. However, this misclassification would not account for all the excess NMSC deaths. Although our data lack treatment information, we divided the follow-up time into three periods, <1, 1–9 and >9 years. The period >9 years of follow-up may reveal the effects due to chemotherapy at most solid tumour sites (Travis *et al*, 1991, 1993; Swerdlow *et al*, 1997, 2000) and we did not find that risks increased during this later follow-up period.

Our findings provide evidence of an increased risk of cancer, particularly melanoma and NMSC, in patients living with CLL in Australia. Chronic lymphocytic leukaemia patients also had an increased risk of death due to melanoma and NMSC, suggesting these skin cancers may be more aggressive in CLL patients. We speculate that a shared risk factor, such as general immune suppression, modulated by UVR exposure may explain the increased risk of melanoma and NMSC in CLL patients. Australia is an area of high solar radiation that makes this particular relationship extremely important (Siskind *et al*, 2002). Information from this study could be used to develop surveillance recommendations for doctors and patients in order to prevent morbidity and mortality. Further research in this area is necessary to understand the true risk of skin cancers and all other second cancers following CLL in Australia. An accurate understanding of the risk of second cancer may lead to changes in clinical practise with regard to the follow-up of CLL patients, such as specific protocols for early detection and treatment of cancers.

## Figures and Tables

**Table 1:** Characteristics of CLL cohort

**Characteristics**	**Incidence study**	**Mortality study**
*Number of CLL patients*	*13 580*	*13 734*
Male	8062	8168
Female	5518	5566
		
Person-years at risk	75 878	84 482
Median age at diagnosis (years)	69	69
Median length of follow-up (years)	4.3	4.9
Number of second primary cancers	1793	—
Percent with second malignancy	13.1%	—
Median interval between first and second diagnosis (years)	3.9	—
Total deaths	—	8246
Non-cancer causes of death (%)	—	35.2
Cancer causes of death (%)	—	64.8

Abbreviation: CLL=chronic lymphocytic leukaemia.

**Table 2:** Site-specific risk of second primary malignancy following CLL

	**Men**			**Women**			**Total**		
**Second cancer site (ICD-10)**	**O**	**SIR**	**95% CI**	**O**	**SIR**	**95% CI**	**O**	**SIR**	**95% CI**
Lip (C00)	33	**6.26**	**4.31, 8.79**	12	**6.80**	**3.51, 11.9**	45	**7.17**	**5.23, 9.60**
Oral cavity and pharynx (C01–C14)	29	**2.30**	**1.54, 3.30**	6	1.46	0.54, 3.17	35	**2.33**	**1.63, 3.25**
Oesophagus (C15)	12	1.05	0.54, 1.83	9	1.65	0.76, 3.14	21	1.34	0.83, 2.05
Stomach (C16)	28	1.20	0.80, 1.74	7	0.74	0.30, 1.52	35	1.17	0.81, 1.62
Colorectal (C18–C20; C218)	146	**1.82**	**1.54, 2.14**	101	**2.02**	**1.64, 2.45**	247	**1.98**	**1.74, 2.25**
Liver (C22)	9	1.14	0.52, 2.16	0	—	—	9	0.97	0.44, 1.84
Gallbladder (C23–C24)	7	1.48	0.59, 3.05	6	1.19	0.44, 2.58	13	1.31	0.70, 2.24
Pancreas (C25)	21	1.17	0.72, 1.79	14	1.00	0.55, 1.68	35	1.12	0.78, 1.56
Larynx (C32)	13	**2.01**	**1.07, 3.44**	0	—	—	13	**2.25**	**1.20, 3.85**
Lung (C33–C34)	216	**1.98**	**1.72, 2.26**	59	**1.86**	**1.41, 2.40**	275	**2.17**	**1.93, 2.45**
Melanoma (C44; M872–M879)	201	**7.40**	**6.42, 8.50**	71	**6.40**	**5.00, 8.07**	272	**7.74**	**6.85, 8.72**
Connective tissue and peripheral nerves (C47, C49)	13	**5.71**	**3.04, 9.77**	4	3.26	0.89, 8.35	17	**5.13**	**2.99, 8.21**
Female breast (C50)	—	—	—	86	**1.93**	**1.55, 2.39**	86	**1.93**	**1.55, 2.39**
Uterine corpus (C54)	—	—	—	26	**3.51**	**2.29, 5.15**	26	**3.51**	**2.29, 5.15**
Ovary (C56)	—	—	—	15	1.52	0.85, 2.51	15	1.52	0.85, 2.51
Prostate (C61)	268	**1.94**	**1.71, 2.18**	—	—	—	268	**1.94**	**1.71, 2.18**
Kidney (C64–C66, C68)	42	**2.93**	**2.11, 3.96**	11	1.50	0.75, 2.69	53	**2.60**	**1.95, 3.40**
Bladder (C67)	58	**2.21**	**1.68, 2.85**	13	1.87	0.99, 3.20	71	**2.42**	**1.89, 3.05**
Eye (C69)	9	**8.55**	**3.91, 16.2**	0	—	—	9	**5.98**	**2.73, 11.4**
Brain (C71–72)	10	1.16	0.56, 2.13	9	1.87	0.85, 3.55	19	1.47	0.89, 2.30
Thyroid (C73)	3	2.87	0.59, 8.37	6	**4.96**	**1.82, 10.8**	9	**3.89**	**1.78, 7.38**
Non-Hodgkin's lymphoma (M967–M972)	3	**0.22**	**0.05, 0.65**	3	0.35	0.07, 1.03	6	**0.28**	**0.10, 0.62**
Hodgkin's disease (M965–M966)	7	**7.59**	**3.05, 15.7**	4	**7.59**	**2.07, 19.4**	11	**7.98**	**3.98, 14.3**
Myeloid leukaemia (M984–M993)	3	0.34	0.07, 1.01	2	0.43	0.05, 1.54	5	**0.40**	**0.13, 0.92**
All LHNs (C81–C96)	15	**0.31**	**0.17, 0.51**	11	**0.38**	**0.19, 0.68**	26	**0.35**	**0.23, 0.51**
All malignant neoplasms (C00–C80)	1265	**2.12**	**2.00, 2.24**	528	**1.84**	**1.69, 2.01**	1793	**2.17**	**2.07, 2.27**

Abbreviations: CI=confidence interval; CLL=chronic lymphocytic leukaemia; ICD=International Classification of Diseases; LHN=lymphohematopoietic neoplasm; O=observed number of cases; SIR=standardised incidence ratio.

Values in bold=95% CI did not include 1.00.

**Table 3:** Site-specific risk of second cancer following CLL by age at first diagnosis and follow-up time

	**Age at CLL diagnosis** a						**Year(s) after CLL diagnosis**					
	**40–54**		**55–64**		**>64**		**<1**		**1-9**		**>9**	
**Second cancer site (ICD-10)**	**O**	**SIR**	**O**	**SIR**	**O**	**SIR**	**O**	**SIR**	**O**	**SIR**	**O**	**SIR**
Lip (C00)	11	**56.5**	9	**8.83**	25	**4.95**	2	3.83	31	**6.61**	12	**16.5**
Colorectal (C18–C20; C218)	12	**2.32**	60	**2.58**	175	**1.82**	27	**2.72**	182	**2.01**	38	**1.98**
Lung (C33–C34)	19	**2.70**	87	**2.78**	169	**1.92**	25	**2.51**	191	**2.08**	59	**2.43**
Melanoma (C44; M872–M879)	32	**22.0**	70	**11.0**	169	**6.20**	17	**6.11**	206	**8.04**	49	**7.51**
Female Breast (C50)	9	**4.41**	21	**3.04**	56	**1.58**	8	2.22	64	**1.96**	14	**2.38**
Uterine corpus (C54)	0	—	7	**5.39**	2	**3.25**	1	1.67	21	**3.86**	4	**9.77**
Prostate (C61)	13	**4.54**	70	**3.02**	184	**1.64**	38	**3.47**	171	**1.67**	59	**2.38**
Kidney (C64–C66, C68)	7	**7.11**	15	**3.48**	31	**2.06**	8	**5.01**	36	**2.43**	9	**4.29**
Bladder (C67)	1	1.38	15	**3.17**	55	**2.30**	6	**2.49**	47	**2.19**	18	**3.61**
All malignant neoplasms (C00–C80)	140	**3.84**	467	**2.89**	1183	**1.89**	163	**2.50**	1270	**2.11**	360	**2.25**

Abbreviations: CLL=chronic lymphocytic leukaemia; ICD=International Classification of Diseases; O=observed number of cases; SIR= standardised incidence ratio.

a Age group <40 years was excluded from table because of small numbers.

Values in bold=95% confidence interval did not include 1.00; cancer types must have been significant (95% confidence interval did not include 1.00) and have at least 20 cases to be included.

**Table 4:** Major contributions to cancer causes of death

**Causes of mortality**	**ICD9–ICD10 codes**	**Percent (% *n*=8246)**
*All cancer deaths*	140–208; C00–C97	64.8
All LHNs	200–208; C81–C96	50.0
All cancer deaths excluding LHNs	140–199; C00–C80	14.8
Lung cancer	162–163; C33–C34	3.2
		
All skin cancer	172–173; C43–C44	3.0
Non-melanoma skin cancer	173; C44–C44	1.9
Melanoma	172; C43–C43	1.1
		
Colorectal cancer	153–154; C18–C21	1.6
Breast cancer	174; C50	0.3
Prostate cancer	185; C61	0.3
		
All non-cancer deaths	All excluding 140–208; C00–C97	35.2

Abbreviations: ICD=International Classification of Diseases; LHN=lymphohematopoietic neoplasm.

**Table 5:** Age standardised cancer mortality ratios for CLL cohort

	**Men**			**Women**			**Total**		
**Causes of mortality**	**O**	**SMR**	**95% CI**	**O**	**SMR**	**95% CI**	**O**	**SMR**	**95% CI**
*All cancer deaths*	3368	**5.99**	**5.79, 6.20**	1974	**7.15**	**6.84, 7.48**	5342	**6.81**	**6.63, 7.00**
All cancer deaths excluding LHNs	882	**1.72**	**1.61, 1.84**	335	**1.36**	**1.22, 1.51**	1217	**1.72**	**1.62, 1.82**
All LHNs	2486	**49.1**	**47.2, 51.1**	1639	**55.0**	**52.4, 57.8**	4125	**54.0**	**52.3, 55.6**
Colorectal cancer	88	**1.32**	**1.06, 1.62**	47	**1.15**	**0.84, 1.52**	135	**1.31**	**1.10, 1.55**
Lung cancer	207	**1.54**	**1.34, 1.77**	60	**1.62**	**1.23, 2.08**	267	**1.75**	**1.55, 1.98**
Breast cancer	0	—	—	26	**0.69**	**0.45, 1.01**	26	**0.69**	**0.45, 1.01**
Prostate cancer	24	**0.64**	**0.41, 0.95**	0	—	—	24	**0.64**	**0.41, 0.95**
									
All skin cancer	186	8.28	7.13, 9.55	58	7.32	5.56, 9.46	244	8.89	7.81, 10.1
Melanoma	61	**4.13**	**3.16, 5.31**	26	**4.94**	**3.23, 7.23**	87	**4.79**	**3.83, 5.90**
Non-melanoma skin cancer	125	**16.2**	**13.5, 19.3**	32	**12.0**	**8.22, 17.0**	157	**17.0**	**14.4, 19.8**
									
All non-cancer deaths	1617	**1.14**	**1.08, 1.19**	1287	**1.14**	**1.08, 1.20**	2904	**1.18**	**1.14, 1.23**
All causes	4985	**2.51**	**2.44, 2.59**	3261	**2.32**	**2.24, 2.40**	8246	**2.55**	**2.49, 2.60**

Abbreviations: CI=confidence interval; CLL=chronic lymphocytic leukaemia; LHN=lymphohematopoetic neoplasm; O=observed; SIR=standardised mortality ratio.

Values in bold=95% CI did not include 1.00.
